# Effect of the Healthy Schools Program on Prevalence of Overweight and Obesity in California Schools, 2006–2012

**DOI:** 10.5888/pcd12.150020

**Published:** 2015-05-21

**Authors:** Kristine A. Madsen, Carolyn Cotterman, Pat Crawford, JoAnn Stevelos, Abbie Archibald

**Affiliations:** Author Affiliations: Carolyn Cotterman, School of Public Health, University of California Berkeley, Berkeley, California; Pat Crawford, University of California Berkeley, Atkins Center for Weight and Health, Berkeley, California; JoAnn Stevelos, Abbie Archibald, Alliance for a Healthier Generation, New York, New York.

## Abstract

**Introduction:**

The Alliance for a Healthier Generation’s Healthy Schools Program (HSP) is a national evidence-based obesity-prevention initiative aimed at providing the schools in greatest need with onsite training and technical assistance (TTA) and consultation with national experts (HSP national advisors) to create sustainable healthy change in schools’ nutrition and physical activity environments. The objective of this study was to evaluate the impact of HSP on the prevalence of overweight and obesity in California schools, from HSP’s inception in 2006 through 2012.

**Methods:**

We used statewide body mass index (BMI) data collected annually from 5th-, 7th-, and 9th-grade students to determine whether enrolling in the HSP’s onsite intervention reduced the prevalence of overweight and obesity in intervention schools (n = 281) versus propensity-score matched control schools (n = 709) and whether increasing exposure to the program (TTA and contact with HSP national advisors) was associated with reductions in the prevalence of overweight and obesity.

**Results:**

Analyses showed no difference between HSP schools and control schools in overweight or obesity prevalence. However, program exposure varied widely among participating schools, and each additional contact with TTA or HSP national advisors was associated with a 0.3% decline in overweight and obesity prevalence (*P* < .05).

**Conclusion:**

HSP appears to be an important means of supporting schools in reducing obesity. Although participation in HSP alone was not sufficient to improve weight status in California schools, there was a clear dose–response relationship to the program. HSP serves as an effective model for addressing childhood obesity among engaged schools.

## Introduction

Obesity is a pressing public health issue. Approximately one-third of young people are overweight or obese (1). Obesity increases risk for many of the leading causes of illness and death, including type 2 diabetes and hypertension (2,3).

Schools have been identified by the Centers for Disease Control and Prevention (CDC), the Institute of Medicine, the White House, and the Office of the Surgeon General as a focal point for obesity prevention (4–8). With more than 95% of young Americans aged 5 to 17 years enrolled in schools (9), educational institutions have the potential to reach virtually all children, including those at highest risk of obesity. The CDC’s evidence-based School Health Guidelines emphasize changing school policy to improve the nutrition and physical activity environments as the most effective means to promote healthier schools (10). Recent cluster-randomized trials of school-based interventions aligned with the CDC Guidelines have demonstrated reductions in children’s weight (11,12).

Despite evidence that coordinated school-based strategies can reduce obesity, much work remains in finding mechanisms to promote wide-scale adoption of such strategies. Schools lack expertise in obesity prevention strategies and have many competing educational priorities that limit their ability to focus on health promotion, particularly in underresourced schools in low-income neighborhoods (13). Experts have highlighted the need for a replicable model to engage schools in the process of change, particularly schools with students at greatest risk for obesity (6,14,15).

The Alliance for a Healthier Generation (the Alliance), founded by the American Heart Association and the William J. Clinton Foundation, created the Healthy Schools Program (HSP) in 2006 with funding from the Robert Wood Johnson Foundation. HSP aims to support schools in creating environments in which physical activity and healthy eating are encouraged and accessible before, during, and after school. HSP sets forth a series of 6 structured steps (6-Step Process), a circular journey that schools continuously take to do the following: 1) convene and maintain a school wellness council, 2) assess the school’s current efforts through completion of the HSP 50-item assessment, 3) develop an action plan based on what is important and achievable in the school community, 4) identify resources to help facilitate implementation, 5) implement evidence-based policies and programs, and 6) celebrate success and monitor progress. Recent studies demonstrated that schools significantly increased their implementation of health-related policies after exposure to HSP (16) and that the amount of TTA was significantly associated with school progress (17). However, no evaluation to date has examined the impact of onsite HSP on the prevalence of obesity in participating schools.

We sought to determine the impact of onsite HSP on childhood obesity from HSP’s inception in 2006 through 2012 by using student body mass index (BMI) data, which are routinely collected in California public schools in grades 5, 7, and 9. More than 5,000 schools across the United States participated in onsite HSP from 2006 through 2011. Although only 6% of schools were located in California, as the largest state in the nation and with robust BMI data, California offers an important vantage point from which to study those most at risk of developing weight-related morbidity. We examined HSP’s impact on California students’ weight by comparing schools that enrolled in HSP with propensity-score matched control schools and by examining dose–response effects among enrolled schools.

## Methods

### Design

We used a propensity-score matched control design and a dose–response analysis to examine the effect of onsite HSP participation on child weight status defined as the prevalence of obesity and overweight and standardized BMI. The study was designated as exempt by University of California Berkeley’s Committee for the Protection of Human Subjects.

### Study sample

**School selection.** In accordance with HSP practice, schools were recruited by a program manager, an Alliance staff member who helped guide schools through HSP’s 6-step process. From 2006 through 2012, Alliance program managers were located in northern and southern California. Within their geographic region, each program manager preferentially targeted school districts with at least 50% of students eligible for free or reduced-price meals (FRPM), selecting districts from urban, suburban, and rural regions. The Alliance estimated that 80% of schools recruited by program managers in California agreed to participate (17); however, no systematic records of recruitment refusals were kept before 2011. Schools that were successfully recruited signed a memorandum of understanding (MOU). HSP data records show that 325 California schools signed an MOU from April 2006 through December of 2011. Analyses were limited to the 281 public schools with BMI and demographic data available. Of the 6,350 non-HSP California schools with similarly complete data, 709 control schools were selected by using propensity-score “nearest neighbor” matching (18) based on the school’s obesity rate in 2006 (baseline), grade level, urbanicity (ie, city, suburb, town, or rural), total school enrollment, FRPM eligibility, and sex and racial/ethnic composition (Table 1). Matches for each year of participation were sought both outside and within HSP districts, excluding schools that had already signed an HSP MOU. Thus, schools that ultimately participated in HSP could have been controls in years before participation. Data on total school enrollment, racial/ethnic composition, urbanicity, and total number of students eligible for FRPM were obtained from the California Department of Education (CDE) website (19).

**Table 1:** Characteristics of California Schools Participating in the Healthy Schools Program, 2006–2011

Characteristic	HSP Schools	Propensity-Score–Matched (Control) Schools	All Other California Schools
**Number**	281	709	6,350
**Urbanicity, %**			
City	38	40	42
Suburb	40	36	38
Town	12	12	7
Rural	10	12	13
**School enrollment, n (SD)**	748 (458)	743 (511)	688 (469)
**FRPM eligibility, % (SD)**	66 (27)	65 (27)	52 (29)
**Race/ethnicity, % (SD)**			
Latino	56 (31)	55 (30)	45 (29)
African American	8 (15)	8 (13)	7 (11)
Asian	17 (24)	17 (23)	9 (14)
Non-Hispanic white	13 (15)	14 (17)	32 (27)
**Female, % (SD)**	49 (6)	49 (6)	49 (7)
**Weight status at baseline, 2006^a^**			
Prevalence of overweight, % (SD)	43 (12)	43 (11)	39 (12)
Prevalence of obesity, % (SD)	23 (9)	23 (9)	21 (10)
BMI z-score, mean (SD)	.71 (.31)	.71 (.30)	.63 (.34)
**Number of observations (school/grade/y combinations)**	853	806	36,973

Abbreviations: FRPM, free or reduced-price meals; HSP, Healthy Schools Program; SD, standard deviation.

a Overweight: BMI ≥85th percentile but <95th percentile; obesity: BMI ≥95th percentile.

**Student-level data.** Student-level fitness data were obtained from the CDE. California schools conduct the multicomponent Fitnessgram assessment (20) among 5th, 7th, and 9th grade students annually in the spring and provide the data to CDE. Data records include student grade, age (years), sex, height (inches), weight (pounds), and race/ethnicity (African American, American Indian/Alaska Native, Asian, Hispanic/Latino, Pacific Islander, or non-Hispanic white). BMI data were available for 84% of 5th, 7th, and 9th grade students enrolled during the study period: 6% of students had no Fitnessgram record; 10% were missing the BMI component of the Fitnessgram; and fewer than 1% had biologically implausible BMI values according to the CDC SAS protocol (21). Similar BMI data were available for HSP and non-HSP schools.

### Intervention

Each HSP program manager worked with an average of 120 schools at a time. Over a 4-year period, program managers provided 9 onsite core workshops related to the 6-Step Process. Additional in-person or telephone TTA was scheduled as needed to support schools, including assistance accessing over 600 online resources, completing the HSP assessment or action plan, or applying for an HSP National Healthy Schools Award. Program managers also arranged in-person or virtual trainings with HSP national advisors to support schools with content-specific material related to the following: 1) school health and school policies and environment, 2) health education, 3) physical education and other physical activity programs, 4) nutrition services, 5) health promotion for staff and 6) family and community involvement. Schools received no funding for participating in HSP.

### Healthy Schools Program exposure

To align with timing of BMI data collection (annually in the spring), schools were considered “exposed” to HSP in the spring following the calendar year in which they signed their MOU. Thus, if a school signed an MOU in 2011, they would be considered exposed as of spring 2012 when the subsequent round of BMI data was collected. Because 2012 was the last year for which BMI data were available, schools signing MOUs in 2011 were the last “cohort” included. This classification means that when a school was considered “exposed” in terms of BMI outcomes, they had a signed MOU for at least 3 months and not more than 14 months. This approach allowed baseline BMI (BMI from the year in which schools signed their MOU) to be included as a covariate. Because HSP is designed to initiate lasting structural change, once a school was categorized as exposed, it was considered exposed in all subsequent years.

In dose–response analyses, duration (cumulative years of exposure) and level of participation for TTA and HSP national advisors combined were considered as independent variables. To determine if one type of contact was more influential than another, TTA and HSP national advisor contacts were considered separately as well as in aggregate.

### Statistical analyses

We used mixed effects linear regression to examine the impact of HSP on 3 different outcomes: percentage of students with a BMI ≥85th percentile but <95th percentile for sex and age (referred to here as overweight); percentage with a BMI ≥95th percentile for sex and age (referred to here as obese); and students with average BMI *z*-score (calculated in SAS Version 9.2 [SAS Institute Inc] using the CDC’s program (22) based on the 2000 sex-specific BMI-for-age growth charts). In the matched control analyses, the predictor was exposure to HSP, and we compared exposed schools with propensity-score–matched controls. In dose–response analyses among the 281 HSP-exposed schools, predictors in separate models were years of TTA exposure, number of TTA contacts, years of exposure to an HSP national advisor, and number of contacts with an HSP national advisor. To enable adjustment for grade level, separate outcomes were created for each grade (5th, 7th, and 9th) at each school. Models were adjusted for data year, grade level, FRPM, sex, race/ethnicity, and baseline weight status from 2006 (to allow adjustment for baseline weight status, observations were restricted to 2007 and later), and school and district effects were included to estimate the average treatment effect.

## Results

Of the 281 schools that signed an MOU by December 2011, 227 (81%) participated in at least some TTA, and 90% of those participated in at least 1 session per year during their years of participation. Participation among early cohorts was lower than for cohorts in later years (Table 2; test-for-trend *P *value < .001). No school had records demonstrating attendance at all 9 core workshops. Only 42 schools (19%) had contact with HSP national advisors (Table 3). Schools participating in TTA had slightly higher enrollment of Asian students than schools with no TTA participation; there were no significant differences in weight status or FRPM eligibility (Table 3). Schools with some HSP national advisor contact had higher Latino but slightly lower African-American enrollment than did schools with no HSP national advisor contact and higher prevalence of overweight and obesity (Table 3).

**Table 2:** Cumulative Median [Interquartile Range] Number of Contacts With the Healthy Schools Program,^a^ by Year Memorandum of Understanding Signed by California Schools, 2006–2011

Cohort	2006	2007	2008^b^	2009	2010	2011
No. of schools	26	66	1	69	49	70
Years (interquartile range) since memorandum of understanding signed						
1	3 (3–3)	1 (0–2)	5	4 (4–5)	4 (3–5)	3 (1–4)
2	3 (3–3)	2 (0–2)	8	6 (5–7)	6 (5–7)	—
3	3 (3–3)	2 (0–3)	16	7 (5–8)	—	—
4	3 (3–3)	2 (0–3)	16	—	—	—

Abbreviation: —, not applicable.

a Includes combined contacts with Healthy Schools Program national advisors and training and technical assistance.

b Values in 2008 represent those for a single school entered the study.

**Table 3:** Characteristics at Baseline of California Schools (N = 281) Participating in the Healthy Schools Program, by Level of Exposure, 2006–2011

Characteristic^a^	Type of Exposure					
	Training and Technical Assistance			Healthy Schools Program National Advisor		
	No Exposure, N = 54	Some Exposure,^b^ N = 227	*P *Value^c^	No Exposure, N = 239	Some Exposure,^b^ N = 42	*P *Value^c^
**School type, n (%)**						
Elementary	41 (76)	135 (59)	.18	151 (63)	25 (60)	.92
Middle	10 (19)	36 (16)	.71	43 (18)	3 (7)	.14
High	3 (6)	34 (15)	.10	36 (15)	1 (2)	.03
K-8 (grades 5 and 7)	0	22 (10)	.01	9 (4)	13 (31)	<.001
**School enrollment, mean (SD)**	795 (415)	804 (562)	.30	830 (563)	644 (302)	.07
**Free or reduced-price meals eligibility**	63 (38)	66 (27)	.40	64 (31)	74 (18)	.23
**Race/ethnicity**						
Latino	57 (34)	54 (28)	.41	53 (30)	65 (23)	.01
African American	9 (13)	9 (14)	.80	9 (14)	6 (8)	.04
Asian	8 (16)	15 (21)	.01	14 (20)	13 (20)	.82
Non-Hispanic white	23 (28)	17 (18)	.81	19 (21)	11 (12)	.15
**Female**	50 (5)	49 (60)	.82	49 (5)	50 (7)	.51
**Weight status**						
Prevalence of overweight^d^	41 (14)	44 (11)	.39	42 (12)	47 (11)	.04
Prevalence of obesity^d^	22 (10)	24 (9)	.41	23 (9)	27 (9)	.02
BMI *z* score, mean (SD)	.66 (.37)	.74 (.28)	.35	.72 (.30)	.79 (.29)	.27

Abbreviation: BMI, body mass index (kg/m^2^).

a Values are % (SD) unless otherwise indicated.

b Some exposure means at least one contact with the Healthy Schools Program National advisors or training and technical assistance.

c *P* values, comparing no exposure with some exposure, correspond to a binomial probability test for school type and to the Mann–Whitney rank sum test for other factors.

d Overweight: BMI ≥85th percentile but <95th percentile; obesity: BMI ≥95th percentile.

Compared with propensity-score matched controls, HSP schools showed no relative reduction in the prevalence of overweight (coefficient, −0.3%; 95% confidence interval [CI], −1.2% to 0.6%) or obesity (coefficient 0.1%; 95% CI, −0.8% to 1.0%), or in mean BMI *z*-score (coefficient −0.01 units; 95% CI, −0.04 to 0.02), in appropriately adjusted analyses.

Among the 281 HSP schools, there was a trend toward decreased overweight (−0.48%, *P* = .09) and obesity (−0.42%, *P* = .08) with each additional year of exposure to (ie, duration of) onsite HSP (Table 4). Looking separately at duration of TTA versus HSP national advisor exposure, each additional year of TTA exposure yielded a trend toward a decline in overweight of 0.49% (*P* = .08) and obesity of 0.44% (*P* = .07), whereas each additional year of exposure to HSP national advisors was associated with larger declines: 1.96% decline in the prevalence of overweight (*P* = .001), 1.73% decline in obesity (*P* = .001), and a 0.04-unit decrease in mean BMI *z*-score (*P* = .02) (Table 4). In models looking at dose rather than duration of program exposure, overweight and obesity declined by 0.3% with each additional contact with the program (TTA and HSP national advisor combined, *P* = .046 and *P* = .014 for overweight and obesity outcomes, respectively). Looking separately at types of dose, only contact with HSP national advisors reached significance, and only for reduction in obesity (Table 4).

**Table 4:** Dose–Response of Exposure to Training and Technical Assistance (TTA) and Healthy Schools Program (HSP) National Advisors, Participating California Schools (N = 281),^a^ 2006–2011

Exposure to TTA or HSP Advisors	Outcome Beta (95% CI) (*P* Value)		
	Overweight^b^ , %	Obese^b^, %	BMI *z*-score
**Total, y**	−.48 (−1.03 to .07) (.09)	−.42 (−.90 to .05) (.08)	−.01 (–.03 to .00) (.14)
TTA, y	−.49 (−1.04 to .05) (.08)	−.44 (−.92 to .03) (.07)	−.01 (−.03 to .00) (.12)
HSP national advisor, y	−1.96 (−3.15 to −.77) (.001)	−1.73 (−2.71 to −.75) (.001)	–.04 (−.07 to −.01) (.02)
**Cumulative total sessions/contacts**	–.25 (–.49 to –.00) (.05)	−.26 (−.47 to −.05) (.01)	−.01 (−.01 to .00) (.05)
Cumulative no. of TTA sessions	–.26 (–.59 to .08) (.13)	−.23 (−.52 to .06) (.11)	−.01 (−.02 to .00) (.10)
Cumulative no. of HSP national advisor contacts	−.44 (–.92 to .04) (.08)	−.50 (−.91 to −.11) (.01)	−.01 (−.02 to .00) (.16)

Abbreviation: BMI: body mass index (kg/m^2^).

a Among the 281 participating schools, the unit of analysis was school/grade/year, with 853 observations.

b Overweight: BMI ≥85th percentile; obese: BMI ≥95th percentile.

## Discussion

This study is the first to report results on HSP’s impact on childhood obesity. The Alliance for a Healthier Generation’s onsite HSP is an ongoing investment in school-based obesity prevention by national experts, nonprofit organizations, and schools.

Overall, schools that signed an HSP MOU did not show greater improvement in students’ weight status than matched control schools. This is not surprising given that almost 20% of schools that signed an MOU did not participate in any HSP activities, and among the 80% that did, participation varied widely. The varied participation in HSP among enrolled schools is not unexpected; implementation of school-based interventions varies significantly across sites, even with researchers present (23). An important line of inquiry will be to learn how to motivate schools to participate at higher levels. Among later HSP cohorts, participating schools were making most of the recommended contacts with the program. Given that schools receive no incentives for participating in HSP, it is heartening that schools engage as fully as they do.

Dose–response analyses suggested that greater duration of participation and dose of exposure to HSP were associated with greater improvements in weight status. Dose reflects greater attendance at TTA workshops and contact with HSP national advisors, both designed to support policy changes to improve the school nutrition and physical activity environments. In this study reliable data on policy implementation were not available, but a prior study demonstrated that increasing program exposure was significantly associated with school progress in the 6-Step Process (17). Future studies should carefully monitor policy implementation and explain clearly the school policies with the greatest impact on health.

Exposure to HSP national advisors appeared to have the greatest impact on students’ weight, though this finding may result from selection bias; fewer than 20% of schools attended HSP national advisor sessions. Although selection bias is a concern, it is reassuring that schools with greatest exposure were those with greatest need, as evidenced by slightly higher prevalence of overweight and obesity than schools with no exposure to HSP national advisors. Nonetheless, the schools that sought the greatest levels of exposure were probably the most motivated, and it is possible that those schools would have done well without the support of HSP. This hypothesis assumes, however, that schools have the expertise and resolve necessary to implement effective obesity-prevention policies without additional guidance. Given schools’ limited financial and staff resources to address multiple curriculum mandates (24), obesity prevention efforts are more likely to be effective with the support of outside experts to both suggest the most effective strategies and to support ongoing motivation for change.

Although the Institute of Medicine is now calling on schools to improve their nutrition and physical activity environments, efforts have long been under way in schools to address obesity. Early interventions such as Planet Health (25) and CATCH (26) were largely curriculum-based with a focus on changing individual behaviors and had modest results. Planet Health demonstrated a decrease in obesity prevalence but only among girls, and CATCH demonstrated no changes in obesity. Pathways, an obesity-prevention intervention among Native American youths, similarly saw no decline in obesity (27). However, the nutrition intervention in the CATCH and Pathways studies was limited to reducing the percentage of calories from fat in school meals rather than altering overall calories or the larger food environment. Both also attempted to improve physical education — CATCH focused on quality and Pathways on quantity, but neither addressed both physical education quality and quantity or other physical activity opportunities in school.

More recently, interventions focused on school policies with broad effects on the nutrition and physical activity environments. The successful School Nutrition Policy Initiative, delivered by the Food Trust, included developing nutritional standards governing all foods sold or served at school and providing training for teachers on integrating physical activity and nutrition into classroom lessons (11). The HEALTHY Study increased the quality and quantity of physical education, and implemented nutrition standards for foods and beverages served throughout school (12). These comprehensive strategies produced a positive effect on weight status (11,12).

HSP, similarly informed by early studies, focuses on changing policy to bring about lasting change to schools’ nutrition and physical activity environments. Notably, this analysis studied real-world implementation of HSP without the potential strengths of implementation under a research protocol. Although prospective research studies are critical to generating evidence, the impact of programs in real-world conditions may not be as great as those seen in rigorous research environments. Because this study reflects HSP executed in the absence of researchers or strict study protocols, exposing schools to HSP is likely to show effects similar to the present results.

Unlike research-grant–funded interventions, onsite HSP is scalable and currently serves 4,500 schools per year. The Alliance now makes HSP tools available on the Internet to any interested school and, over the last few years, worked to create virtual online trainings that mimic the onsite model. Given the potentially greater reach and scalability of an online model, a randomized trial is currently investigating the differential impact between online exposure and the onsite model.

Although using existing data to study the impact of HSP is a strength of this study, additional limitations merit comment. Records of schools that declined to participate in HSP were not available, another source of selection bias; however, this limitation is unlikely to change the null findings in our propensity-score–matched control analysis and would not affect our dose–response analyses. Schools’ implementation of health-related policies at baseline was unknown but could account for some differences seen in dose–response analyses. Temporal trends in obesity may affect our results, although we adjusted for year in all analyses and included propensity-score matched controls. Findings might not generalize beyond California. Because of the rolling nature of enrollment but a fixed period of BMI assessment each spring, actual duration of participation when schools were first considered exposed varied among schools. This would be likely to weaken our ability to detect associations. Additionally, some schools had only 1 year of exposure by 2012, which may not be sufficient time to show an effect on BMI. Fewer than 5% of HSP schools were charter schools; however, charter schools, which may have greater ability to enact changes than traditional schools, are an important population in which to study programs such as HSP. Finally, district or local initiatives not associated with HSP may have been implemented in participating schools and could account for some of the improvements seen in weight status.

Participation in HSP appears to reduce the prevalence of overweight and obesity among students in high-need schools. HSP could serve as an important national model for helping schools to improve their nutrition and physical activity environments.
