# Erratum, Vol. 12, May 21 Release

**DOI:** 10.5888/pcd12.150020e

**Published:** 2015-07-09

In the article “Effect of the Healthy Schools Program on Prevalence of Overweight and Obesity in California Schools, 2006–2012,” an error appeared in the last paragraph of the Results section. The second to last sentence in that paragraph erroneously stated the decline in overweight and obesity as 3%. That sentence should read as follows:

In models looking at dose rather than duration of program exposure, overweight and obesity declined by 0.3% with each additional contact with the program (TTA and HSP national advisor combined, *P* = .046 and *P* = .014 for overweight and obesity outcomes, respectively).

The correction was made to our website on June 2, 2015, and appears online at http://www.cdc.gov/pcd/issues/2015/15_0020.htm. We regret any confusion or inconvenience this error may have caused.

